# Influenza C virus NS1 protein counteracts RIG-I-mediated IFN signalling

**DOI:** 10.1186/1743-422X-8-48

**Published:** 2011-02-02

**Authors:** Karin Pachler, Reinhard Vlasak

**Affiliations:** 1 Department of Molecular Biology, University of Salzburg, Billrothstrasse 11, 5020 Salzburg, Austria

## Abstract

The nonstructural proteins 1 (NS1) from influenza A and B viruses are known as the main viral factors antagonising the cellular interferon (IFN) response, inter alia by inhibiting the retinoic acid-inducible gene I (RIG-I) signalling. The cytosolic pattern-recognition receptor RIG-I senses double-stranded RNA and 5'-triphosphate RNA produced during RNA virus infections. Binding to these ligands activates RIG-I and in turn the IFN signalling. We now report that the influenza C virus NS1 protein also inhibits the RIG-I-mediated IFN signalling. Employing luciferase-reporter assays, we show that expression of NS1-C proteins of virus strains C/JJ/50 and C/JHB/1/66 considerably reduced the IFN-β promoter activity. Mapping of the regions from NS1-C of both strains involved in IFN-β promoter inhibition showed that the N-terminal 49 amino acids are dispensable, while the C-terminus is required for proper modulation of the IFN response. When a mutant RIG-I, which is constitutively active without ligand binding, was employed, NS1-C still inhibited the downstream signalling, indicating that IFN inhibitory properties of NS1-C are not necessarily linked to an RNA binding mechanism.

## Background

Innate immune response is the first unspecific defence against viral infections, in which the induction of type I IFNs is essential for controlling influenza virus replication and spread. Recently, RIG-I has been identified as the major cytosolic pattern-recognition receptor sensing RNA in influenza virus-infected cells, thereby initiating the IFN signalling [1,2]. RIG-I, which belongs to the DExD/H box family of RNA helicases, consists of two N-terminal caspase activation and recruitment domains (CARDs), an internal ATP-dependent RNA helicase domain, and a C-terminal repressor domain that holds the protein in an inactive state [3,4]. Binding of the repressor domain to dsRNA or 5'-triphosphate RNA, at least the latter of which is present in detectable amounts during influenza virus infections [5], induces a conformational change that leads to exposition of the CARDs. Tripartite motif protein 25 (TRIM25) interacts with the first CARD of RIG-I and ubiquitinates the second CARD [6]. Ubiquitinated RIG-I proteins multimerise and form a complex with mitochondrial antiviral signalling adaptor (MAVS), also termed IPS-1/Cardif/Visa. The subsequent signal cascade leads to activation of transcription factors IRF3, IRF7, AFT-2/c-Jun, and NFκB, which translocate to the nucleus to form the IFN-β enhanceosome. The IFN-β expression results in transcription of more than 100 IFN-induced genes, many of which are known to exhibit anti-influenza virus activity (reviewed in [7]).

For influenza A and B viruses, NS1 has been identified as the main antiviral protein antagonising the cellular IFN signalling. The influenza A virus NS1 has been reported to inhibit RIG-I-mediated IFN synthesis [8-10]. This IFN inhibitory property has been discussed to be due to its RNA-binding activity [11,12], which is important for optimal inhibition of type I IFN induction [13,14]. Besides sequestering viral RNA from being detected by RIG-I, NS1-A can also interact with the RIG-I complex independently of an RNA bridge. Expression of NS1-A inhibited IFN induction by a constitutively activated RIG-I protein lacking the helicase and repressor domains [9]. Recently, human TRIM25 protein was identified as an NS1-binding protein too, and NS1-TRIM25 complex formation led to inhibition of RIG-I ubiquitination and consequently its downstream signalling [15]. Earlier studies on the modulation of the IFN-β production by NS1-A indicated that NS1-A inhibits activation of transcription factors NFκB, IRF3, and AFT-2/c-Jun [16-18], obviously as a result of its interference with RIG-I signalling. In addition to antagonising RIG-I-mediated IFN-β expression, NS1-A has been found to inhibit the activity of the IFN-induced antiviral proteins protein kinase R (PKR) and 2'-5'-oligoadenylate synthetase (OAS). Moreover, NS1-A has been shown to bind to components involved in cellular mRNA processing, export, and translation, thereby inhibiting cellular protein synthesis (reviewed in [7]). Like NS1-A, the influenza B virus NS1 protein is essential for the regulation of RIG-I-mediated IFN-β production (reviewed in [7]). In contrast, no reports are available how influenza C virus modulates the immune response.

Influenza C virus harbours seven single-stranded RNA segments of negative polarity, of which the smallest segment, NS, codes for NS1 and, from a spliced mRNA transcript, for nuclear export protein/nonstructural protein 2 (NEP/NS2). The NS1 proteins of influenza C virus strains are commonly composed of 246 amino acids [19]. We have recently investigated that NS1-C from strain C/JJ/50 is in contrast made of only 239 amino acids [20]. Muraki et al. [21] have reported that NS1-C is involved in splicing of viral mRNAs and that it is localised in the nucleus in an early stage of infection, while in later stages of infection it predominantly resides in the cytoplasm. This cytoplasmic localisation may reflect RIG-I antagonising properties of NS1-C.

To elucidate whether NS1 from influenza C virus also counteracts the cellular IFN response, we examined the effect of NS1 expression on the IFN-β promoter activity in HEK-293TN cells using a luciferase-reporter assay.

## Results and Discussion

**Figure 1:**
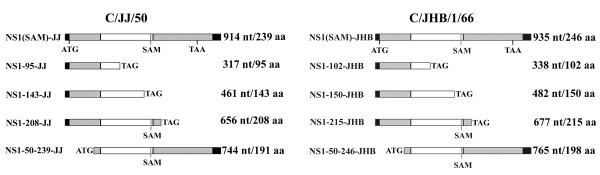
**Overview of NS derivatives constructed from influenza C/JJ/50 and C/JHB/1/66 viruses**. Sequences are shown in positive sense. NS1(SAM)-JJ and NS1(SAM)-JHB have wild type-like sequence lengths but are splice acceptor site-mutated (SAM) to prevent NEP/NS2 expression. Start and stop codons - whether native or introduced by cloning - are depicted. Black bars: noncoding ends. All primer sequences are available upon request.

First, plasmids expressing full-length and truncated NS1 from influenza C virus strains C/JJ/50 and C/JHB/1/66 were generated (Figure 1). pCI-NS1(SAM)-JJ and -JHB code for the entire NS1 proteins from both strains. The NS1 cDNAs were PCR-amplified from templates pPMV-NS and pPMV-NS-C/JHB/1/66 [20]. In order to prevent formation of NEP/NS2, the splice-acceptor sites of NS were mutated via internal primers: For strain C/JJ/50, nucleotide 504, for strain C/JHB/1/66, nucleotide 525 was changed from A to C, which does not affect the amino acid sequences of NS1. The PCR fragments were internally ligated with *BsmBI *sites. The entire NS1 fragments were cloned into vector pCI (Promega) via *NheI *and *XbaI *restriction sites. To construct plasmids for expression of C-terminally truncated NS1 proteins from both virus strains (pCI-NS1-95-JJ and pCI-NS1-102-JHB, pCI-NS1-143-JJ and pCI-NS1-150-JHB, and pCI-NS1-208-JJ and pCI-NS1-215-JHB), the different-sized NS1 fragments were PCR-amplified from the respective pCI-NS1(SAM) templates with reverse primers introducing two stop codons and were cloned into *XhoI *and *XbaI *restriction sites of vector pCI. For generation of plasmids encoding N-terminally truncated NS1 proteins (pCI-NS1-50-239-JJ and pCI-NS1-50-246-JHB), NS1 fragments were PCR-amplified with the respective pCI-NS1(SAM) templates and a forward primer adding a start codon upstream of amino acid 50. The fragments were inserted into pCI with *XhoI *and *XbaI *restriction sites.

In a first set of experiments, 90% confluent HEK-293TN cells in 24-well plates (kindly provided by Dr. Albert Duschl, University of Salzburg), maintained in Dulbecco's modified Eagle's medium with 10% fetal bovine serum and 2 mM L-glutamine, were cotransfected with 500 ng of one of the plasmids encoding full-length NS1 (pCI-NS1[SAM]-JJ or pCI-NS1[SAM]-JHB), or with empty pCI vector as control, together with 250 ng of a reporter plasmid for IFN-β promoter activity (pGL4.10-IFN-β-prom). pGL4.10-IFN-β-prom encodes luciferase under the control of the murine IFN-β promoter. Nucleotides -125 to +19 of the IFN-β promoter, derived from genomic MEF-23-1 DNA, were inserted immediately upstream of the firefly luciferase gene (luc2) of the promoterless vector pGL4.10 (Promega) via *NheI *and *NcoI *restriction sites. In addition, 50 ng of a plasmid expressing β-galactosidase (p4TO/lacZ, kindly provided by Dr. Annemarie Frischauf, University of Salzburg) as internal transfection reference and 50 ng of a RIG-I expression plasmid (pCI-RIG-I) were included. pCI-RIG-I encodes the full-length human RIG-I (925 amino acids). Plasmid pCR-XL-TOPO-BC136610 (purchased from Open Biosystems) served as template to amplify the 2778-nucleotide ORF of RIG-I, which was inserted into vector pCI via *XhoI *and *NotI *restriction sites. Transfections were performed with 2 μl of Lipofectamine 2000 (Invitrogen) in Opti-MEM medium with Glutamax (Invitrogen). Six hours post transfection, the transfection mixes were changed for 1 ml Opti-MEM with Glutamax. 24 hours post transfection, cells were stimulated with 1 μg poly(I:C) (purchased from Sigma-Aldrich) or 800 ng vRNA (isolated from egg-grown influenza C virus using QiaAmp viral RNA mini kit according to Qiagen's instructions, and subsequently digested with DNAseI), again by transfection with 2 μl Lipofectamine 2000. Eight hours post induction, cells were lysed in 80 μl cell culture lysis reagent (Promega), and supernatants were cleared by centrifugation. To determine luciferase activity of cell supernatants, 20 μl of each sample were mixed with Luciferase Assay Reagent (Promega) and measured by a GloMax™ 96 Microplate Luminometer (from Promega, kindly provided by Dr. Arnulf Hartl, Paracelsus Medical Private University, Salzburg) according to the manufacturer's recommendations. As internal transfection reference, β-galactosidase activity (from plasmid p4TO/lacZ) of samples was measured as well: 10 μl of cell supernatants were mixed with 125 μl of Z-buffer (100 mM sodium phosphate, pH 7.4, 10 mM KCl, 1 mM MgSO_4_, 50 mM 2-mercaptoethanol) and 25 μl of ONPG solution (4 mg/ml Ortho-nitrophenyl-β-D-galactopyranoside from Sigma in 100 mM NaPO_4_, pH 7.4). The OD at 415 nm of the samples was determined with Spectra Fluor spectrafluorimeter (Tecan) for three times with one minute delay between every read. The change in OD per minute (ΔOD/min) was calculated. Finally, the value of luciferase activity was divided through the ΔOD/min of β-galactosidase activity to gain the normalised luciferase activity. All transfection assays were performed three times in duplicates.

**Figure 2:**
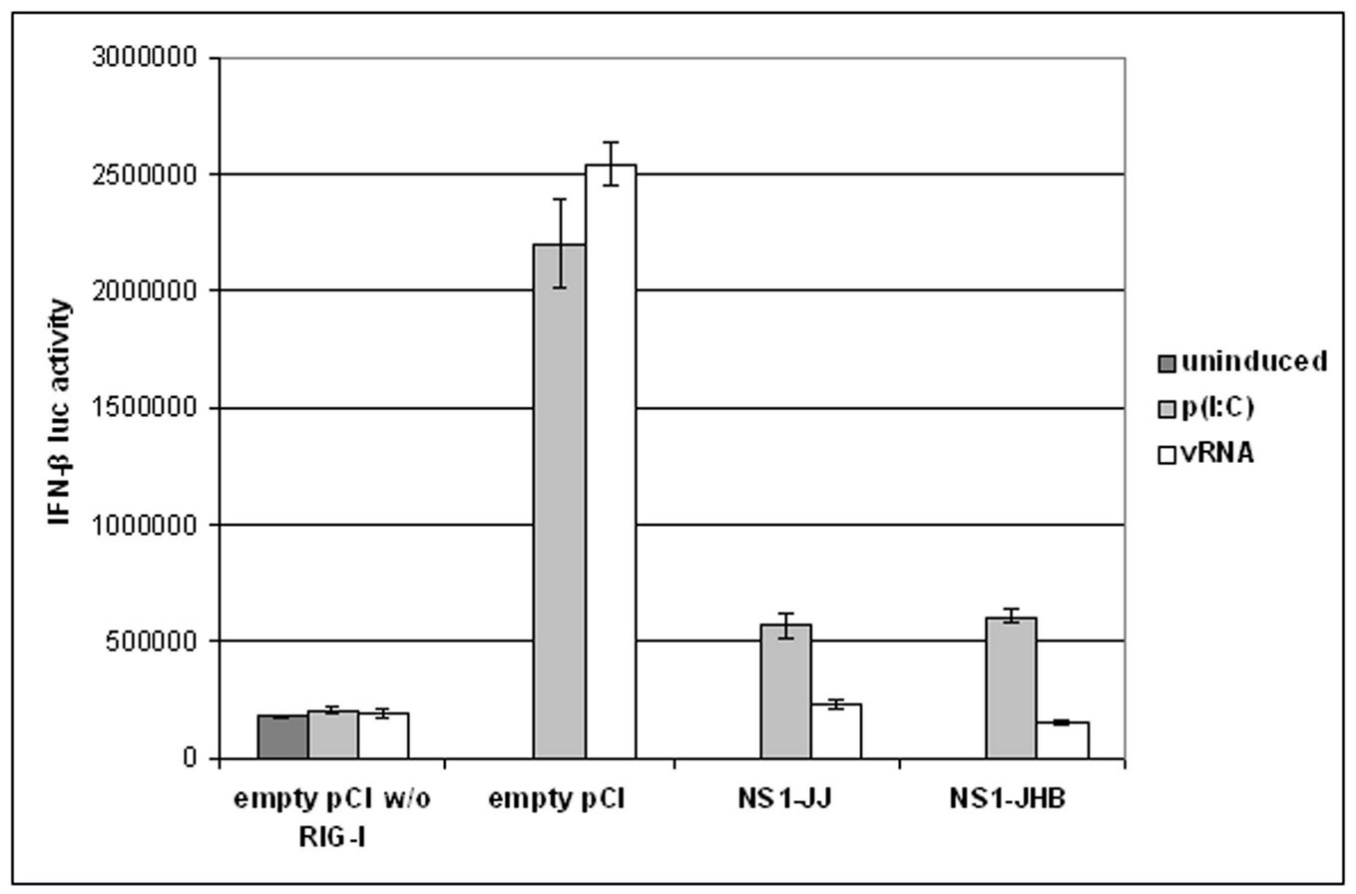
**NS1-C inhibits IFN-β promoter activity induced by RIG-I**. HEK-293TN cells were transiently transfected with IFN-β luciferase reporter plasmid, RIG-I and β-galactosidase expression plasmids, and vectors encoding NS1 from C/JJ/50 or C/JHB/1/66, or empty pCI vector. 24 h post transfection, cells were stimulated with poly(I:C) or vRNA, and eight hours post induction, luciferase activity of cell lysates was measured and normalised to β-galactosidase activity. Data shown are representative for three experiments with transfections performed in duplicates. Error bars indicate standard deviations. The zero control without ectopic RIG-I expression is depicted at the left.

As HEK-293TN cells do not express a pattern-recognition receptor for influenza virus detection, without ectopic RIG-I expression the IFN-β promoter was hardly switched on (Figure 2). In contrast, in cells transfected with RIG-I and stimulated with poly(I:C) or vRNA, the IFN-β promoter was highly activated. With addition of NS1 from either strain C/JJ/50 or C/JHB/1/66, this IFN-β promoter activation was clearly diminished. When cells had been stimulated with the synthetic dsRNA poly(I:C), cotransfected NS1 led to a reduction of reporter activity to one fourth. When cells had been induced with vRNA from influenza C virus, reporter activity even equalled the zero control (without ectopic RIG-I) in the presence of NS1.

**Figure 3:**
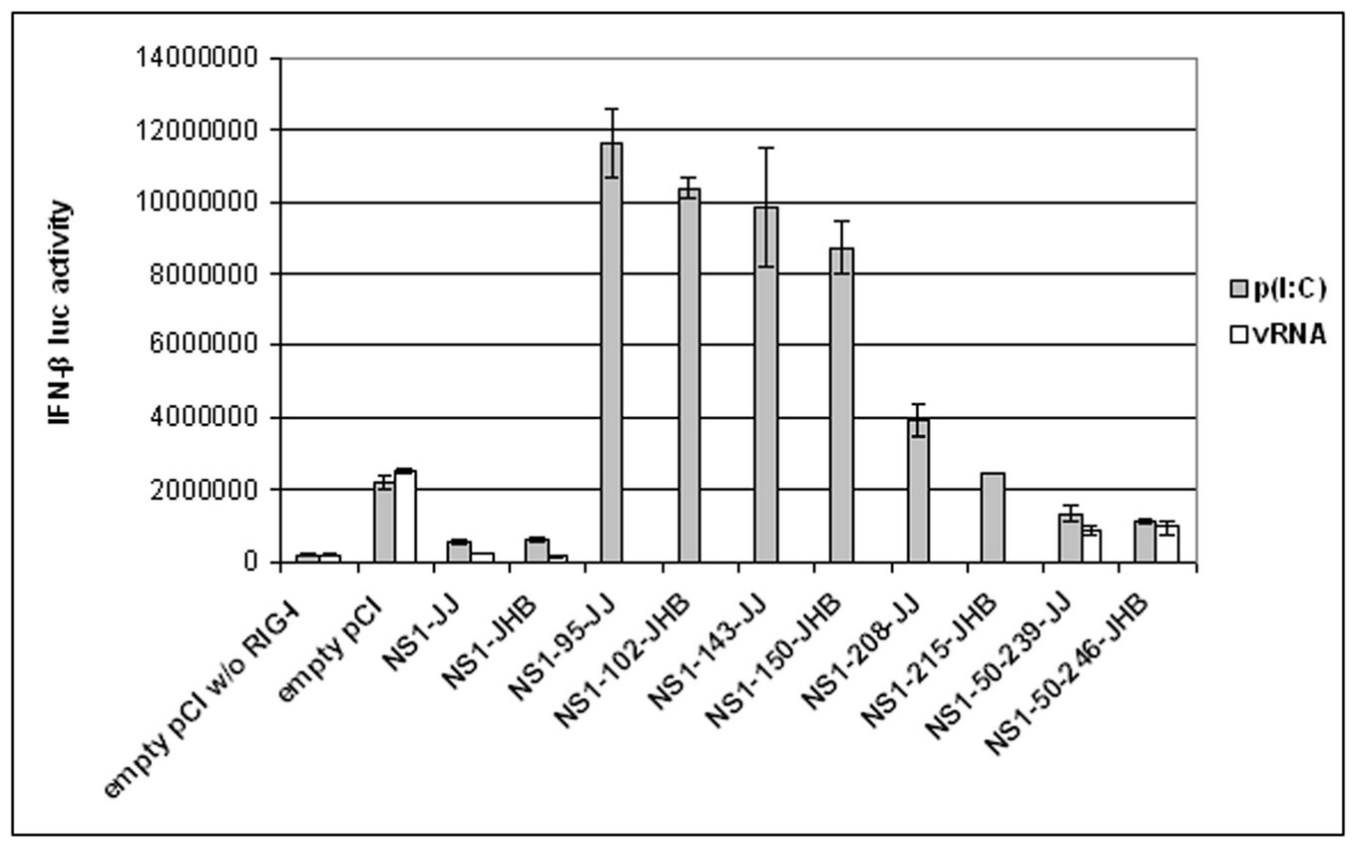
**Impact of truncated NS1 proteins on IFN-β promoter activity**. HEK-293TN cells were transiently transfected with IFN-β luciferase reporter plasmid, RIG-I and β-galactosidase expression plasmids, and vectors encoding full-length or truncated NS1 from C/JJ/50 or C/JHB/1/66, or empty pCI vector. 24 h post transfection, RIG-I was induced with poly(I:C) or vRNA, and eight hours post induction, luciferase activities of cell lysates were measured and normalised to β-galactosidase activities. Only full-length and N-terminally-truncated NS1 proteins inhibited reporter activity. Error bars indicate standard deviations.

Next, the regions of NS1 involved in IFN-β promoter inhibition were determined. HEK-293TN cells were transfected with the IFN-β promoter reporter plasmid, RIG-I plasmid, and β-galactosidase plasmid along with either full-length or truncated NS1 expression plasmids (see Figure 1). In a first round of experiments, RIG-I was induced with poly(I:C). As shown in Figure 3, all C-terminally truncated NS1 proteins did not support IFN-β promoter inhibition. On the contrary, these NS1 derivatives even seemed to stimulate IFN-β promoter activity. The greater the C-terminal deletion, the more strongly the reporter was activated. In contrast, the N-terminally deleted NS1 variants (NS1-50-239-JJ and NS1-50-246-JHB) led to suppression of the IFN-β promoter. But the effect of these shortened NS1 proteins was not as strong as the influence of the full-length isoforms. When RIG-I was induced with vRNA, the IFN-β inhibitory properties of the N-terminally deleted NS1 proteins were strengthened.

**Figure 4:**
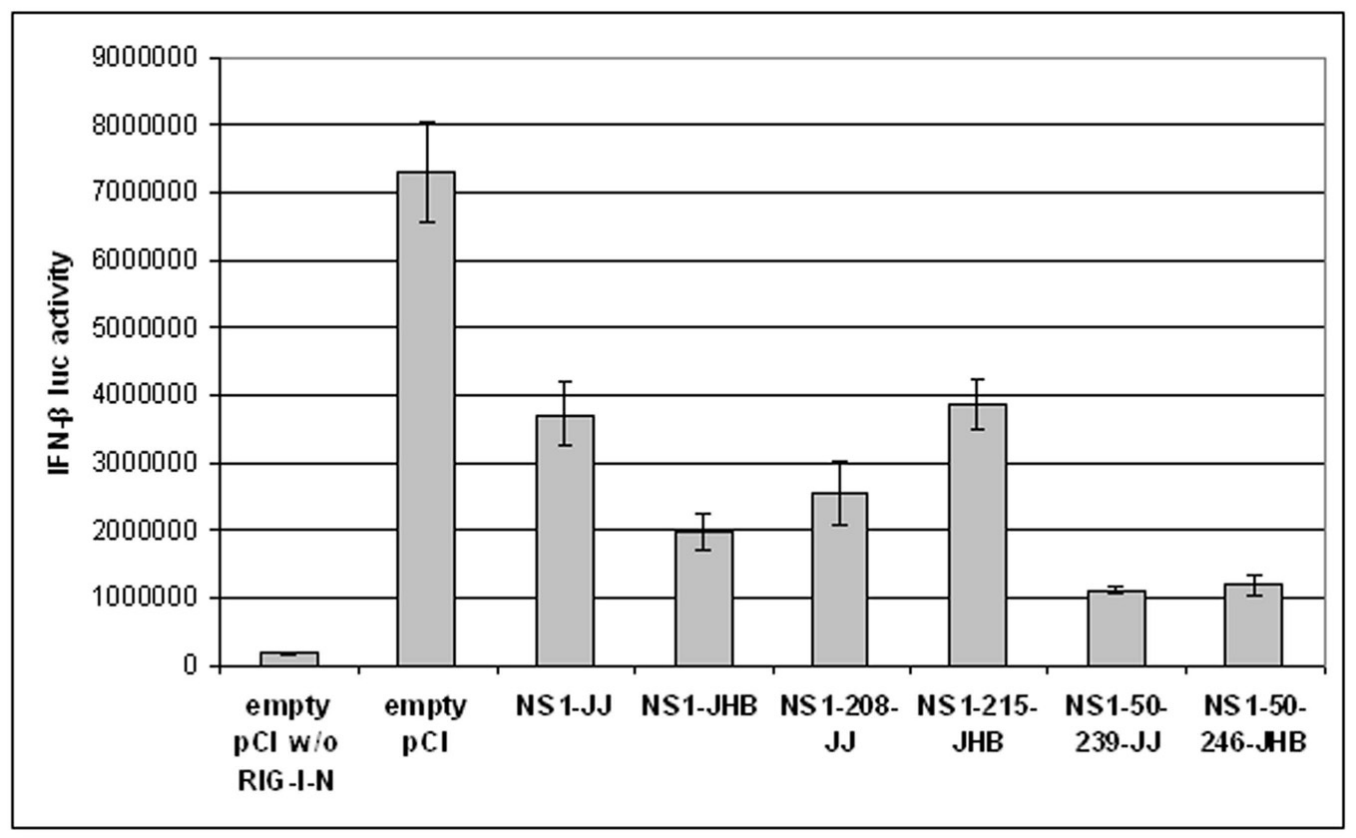
**NS1-C antagonises IFN response in the absence of an RNA stimulus**. HEK-293TN cells were transfected with IFN-β luciferase reporter plasmid, plasmids encoding mutant RIG-I (RIG-I-N) and β-galactosidase, and vectors encoding full-length or truncated NS1 from C/JJ/50 or C/JHB/1/66, or empty pCI vector. Relative luciferase activities of cell lysates 32 hours post transfection were determined. Error bars indicate standard deviations.

To find out whether NS1-C also prevents RIG-I-induced IFN signalling independent of an RNA-binding mechanism, as reported for influenza A virus NS1 [9], reporter-transfection experiments were carried out with a mutant RIG-I protein. RIG-I-N lacks the helicase and repressor domains and is consequently active without RNA stimulation [2]. Vector pCI-RIG-I-N was generated, which codes for the N-terminal 284 amino acids of human RIG-I protein. From template plasmid pCR-XL-TOPO-BC136610, an 858 nucleotides long fragment (including two stop codons introduced by reverse primer) was cloned into vector pCI via *XhoI *and *NotI *restriction sites. Cell transfections were performed as outlined before, with the difference that no RNA was necessary for induction. As shown in Figure 4, transfection of the mutant RIG-I resulted in even elevated IFN-β promoter activity (compare to Figure 2). In the presence of NS1, this IFN-β promoter activation was decreased, whereat NS1 from strain C/JHB/1/66 exhibited stronger inhibitory effects. The N-terminally truncated NS1 proteins from both strains (NS1-50-239-JJ and NS1-50-246-JHB) antagonised the IFN-β promoter activity to an even higher extent. And - most strikingly - even NS1 proteins missing the C-terminal 31 amino acids (NS1-208-JJ and NS1-215-JHB) showed IFN inhibitory properties. All shorter NS1 proteins did not reduce reporter activity, but on the contrary stimulated reporter activity (data not shown). This effect had also been observed in the presence of full-length RIG-I (see Figure 3). These data suggest that NS1-C interacts with components of the IFN signalling independent of an RNA bridge. Whether NS1-C binds to and consequently shields vRNA from being detected by a pattern-recognition receptor remains to be studied in future experiments.

## Conclusion

In conclusion, the results of this study demonstrate that influenza C virus NS1 protein has IFN antagonistic properties like the respective proteins from influenza A and B viruses. Mapping of the NS1 regions involved in inhibition of the IFN-β promoter showed that proteins devoid of the N-terminal 49 amino acids still exhibited IFN antagonistic properties. NS1 proteins missing parts of the C-terminus rather stimulated IFN-β promoter activation. The shorter the NS1 C-terminal portion, the greater the stimulation was observed. Therefore, both N- and C-terminal parts of the full-length NS1-C protein are apparently involved in contacting a component of the RIG-I-mediated signalling, and the C-terminus seems to inhibit downstream signalling. The C-terminally truncated NS1-C proteins lose this inhibitory property, but still seem to contact their counterparts in RIG-I signalling and lead to activation. In contrast, for NS1-A the first 73 amino acids, which harbour RNA-binding activity, were reported to be sufficient for inhibition of the IFN signalling [22]. In case of NS1-B, both the N-terminal 93 amino acids as well as the remaining C-terminus could independently regulate the IFN response [23].

Expression of NS1-C also inhibited the signalling mediated by RIG-I-N, suggesting that NS1-C acts as IFN antagonist independent of an RNA binding mechanism. In the presence of RIG-I-N, the N-terminally deleted NS1-C proteins as well as NS1-C proteins lacking the C-terminal 31 amino acids suppressed the IFN-β promoter-dependent expression. How exactly the NS1-C protein interacts with components of the RIG-I-directed IFN signalling may only be hypothesised at the moment. NS1-C may get in contact with the RIG-I-TRIM25 complex, inhibiting ubiquitination of the RIG-I CARD, as observed for NS1-A. As the ultimate C-terminal amino acids are not required for inhibition of IFN signalling initiated by RIG-I-N, but are necessary for inhibition of RIG-I-mediated signalling, this C-terminus of NS1-C may specifically interact with a C-terminal part of RIG-I or RNA bound to RIG-I. As NS1-C enhances viral mRNA splicing [21], it has obviously RNA-binding activity.

On basis of this work and our recently established system for the rescue of recombinant influenza C viruses [20], NS1-mutated attenuated recombinant influenza C viruses may be created to investigate the roles of NS1 in more detail. Moreover, the generated NS1-truncated plasmids may be employed for determination of the regions of NS1 important for its splicing activity.

## Competing interests

The authors declare that they have no competing interests.

## Authors' contributions

KP designed the study, performed the experiments, analysed the results, and drafted the manuscript. RV supervised all phases of the project: conception and design of the experiments, analysis of the results and writing of the manuscript. All authors read and approved the final manuscript.
